# Metabolites involved in cellular communication among human cumulus-oocyte-complex and sperm during *in vitro* fertilization

**DOI:** 10.1186/s12958-015-0118-9

**Published:** 2015-11-09

**Authors:** María José Gómez-Torres, Eva María García, Jaime Guerrero, Sonia Medina, María José Izquierdo-Rico, Ángel Gil-Izquierdo, Jesús Orduna, María Savirón, Leopoldo González-Brusi, Jorge Ten, Rafael Bernabeu, Manuel Avilés

**Affiliations:** Department of Biotechnology, University of Alicante, 99, Carretera de San Vicente s/n, Alicante, 03016 Spain; Instituto Bernabeu of Fertility and Gynecology, Alicante, 03016 Spain; Research Group on Quality, Safety and Bioactivity of Plant Foods, Food Science and Technology Department, CEBAS-CSIC, Espinardo (Murcia), Spain; Department of Cell Biology and Histology, Faculty of Medicine, University of Murcia, Campus Mare Nostrum, Espinardo 30100 and IMIB, Murcia, Spain; Institute of Materials Science of Aragon, CSIC-University of Zaragoza, 50009 Zaragoza, Spain

**Keywords:** Cumulus cells, Acrosome reaction, Lysophosphatidylcholine, Phytosphingosine, Metabolomics

## Abstract

**Background:**

Fertilization is a key physiological process for the preservation of the species. Consequently, different mechanisms affecting the sperm and the oocyte have been developed to ensure a successful fertilization. Thus, sperm acrosome reaction is necessary for the egg coat penetration and sperm-oolema fusion. Several molecules are able to induce the sperm acrosome reaction; however, this process should be produced coordinately in time and in the space to allow the success of fertilization between gametes.

The goal of this study was to analyze the metabolites secreted by cumulus-oocyte-complex (COC) to find out new components that could contribute to the induction of the human sperm acrosome reaction and other physiological processes at the time of gamete interaction and fertilization.

**Methods:**

For the metabolomic analysis, eighteen aliquots of medium were used in each group, containing: a) only COC before insemination and after 3 h of incubation; b) COC and capacitated spermatozoa after insemination and incubated for 16–20 hours; c) only capacitated sperm after 16–20 h in culture and d) only fertilization medium as control. Six patients undergoing assisted reproduction whose male partners provided normozoospermic samples were included in the study. Seventy-two COC were inseminated.

**Results:**

The metabolites identified were monoacylglycerol (MAG), lysophosphatidylcholine (LPC) and phytosphingosine (PHS). Analysis by PCR and *in silico* of the gene expression strongly suggests that the cumulus cells contribute to the formation of the PHS and LPC.

**Conclusions:**

LPC and PHS are secreted by cumulus cells during *in vitro* fertilization and they could be involved in the induction of human acrosome reaction (AR). The identification of new molecules with a paracrine effect on oocytes, cumulus cells and spermatozoa will provide a better understanding of gamete interaction.

**Electronic supplementary material:**

The online version of this article (doi:10.1186/s12958-015-0118-9) contains supplementary material, which is available to authorized users.

## Background

In the mammalian genital tract, fully mature oocytes that are ready for fertilization are surrounded by a thick vitelline envelope called the *zona pellucida* (ZP), which, in turn is surrounded by numerous cumulus cells embedded in an acellular matrix. Collectively, these are known as the cumulus-oocyte complex (COC).

The *cumulus oophorus* is unique to the egg of eutherian mammals [1]. Cumulus cells are involved in oocyte growth and maturation [2]. However, the role of *cumulus oophorus* during fertilization dependent up on the species [3]. Some studies in mice, have reported that *cumulus oophorus* plays a key role during *in vivo* fertilization and failure in its formation affects this process [4–8] On the other hand, the *cumulus oophorus* is not equally important during *in vitro* fertilization as it is during *in vivo* fertilization. Zhuo et al. (2002) [9] reported that KO mice models that do not form the cumulus matrix (e.g. bikunin) are able to ovulate oocytes, but these oocytes are not fertilized *in vivo*. However, they can be normally *in vitro* fertilized [9], although the reason for this is not fully understood. Other studies revealed the role of cumulus cells in the development of human sperm fertilizing ability, during *in vitro* fertilization in human [10, 11], cattle [12, 13] and pigs [14, 15].

In relation to dialogue between oocyte and spermatozoa, it is known that in human cumulus cells affect various sperm functions [16–20]. Previous studies have used the *cumulus oophorus* for sperm selection in human [17, 21]. Several reports have indicated that progesterone is secreted by the cumulus cells and is able to induce the sperm acrosome reaction (AR) [22–25]. The AR is a process that starts at the cell apex, after which the acrosomal content is released very slowly [26]. The AR is required for the sperm to fuse to the oolema [10]. Other authors have previously indicated that ZP glycoproteins are able to induce the AR in different animal and human models [27, 28]. However, the site where spermatozoa begin their RA is subject to controversy [29]. In hamster, Yanagimachi and Phillips (1984) [30] observed that most spermatozoa *in vivo* initiate their AR while progressing though the cumulus. Other authors maintained that the site where AR begins is the ZP [31–33]. Using *in vitro* fertilization and transgenic mouse spermatozoa, which enable the onset of the AR to be detected using fluorescence microscopy, Jin et al. (2010) [34] found that the spermatozoa that began the AR before reaching the ZP were able to penetrate the ZP and fused with the oocyte´s plasma membrane. Their study suggests a major role for cumulus cells and their matrix during fertilization. Other previous reports reached similar conclusions [35, 36]. However, the precise mechanism responsible for this process remains to be clarified.

The action of cumulus cells on sperm function may be mediated via the secretory products of the cells [37–40]. Although a number of studies have reported that cumulus cells provide soluble factors affecting sperm functions, little is known about the role of the cumulus as a promoting element in fertilization. Only a few candidates have been identified in the conditioned medium as cumulus-derived factors. Prostaglandins, PGE1, PGE2, and PGE2ALFA were detected in the incubation medium of COC [41, 42]. Progesterone was identified as another candidate, which induced hyperactivated flagellar movement and the AR as well [22, 43]. Other studies have demonstrated that in human [44, 45], mouse [46]; pig [47] and rabbit [48] the cumulus cells synthesize and secrete progesterone.

However, molecules other than progesterone may be a factor responsible for these effects. For example, a component that induce the AR is the lysophosphatydic acid (LPA) derivated from lysophosphatidylcholine (LPC) [22,49–52]. The presence of LPA has been described in different biological fluid including the seminal plasma [53, 54] and the follicular fluid [55, 56]. It is well known that the follicular fluid can induce the sperm AR [57–59]. However, its function has been related with the presence of progesterone. After ovulation, some follicular fluid components can be detected in the oviductal fluid leading to speculation concerning a potential role for LPA at the time of fertilization [55].

LPC is another component that shows an ability to induce the AR in human [49] and bovine [50] sperm, with an even higher efficiency than progesterone. Then, it seems that nature have developed several mechanisms by using several molecules to ensure that the important physiological process, AR, take place in the proximity in time and place to allow the success of fertilization between gametes.

The goal of this study was the analysis of the metabolites secreted by COC to identify new cumulus-derived factors that may contribute to the induction of human sperm AR and other physiological processes at the time of gamete interaction and fertilization. A better understanding of the molecular mechanisms involved in the process of fertilization may lead to the development of new pharmacological strategies to treat infertility and for the improvement of assisted reproduction techniques (ARTs) or for a new and more physiological birth control approach.

## Methods

### Patients

This study, approved by the Instituto Bernabeu Institutional Review Board, included six patients enrolled at the Instituto Bernabeu (Alicante, Spain) for assisted reproduction with egg donation, whose male partners showed normozoospermic semen samples according to World Health Organization criteria [60], and where conventional IVF was indicated. Written informed consent was obtained for each patient. In all the cases, the percentages of fertilization were over 25 % (Table 1), which assessment the fertilization capacity sperm in all the samples.

**Table 1:** Percentages of fertilization per case (COC: cumulus-oocyte-complex)

CASE	Number COC	% Fertilization
1	11	81.8
2	12	75
3	12	66.6
4	12	25
5	11	63.6
6	14	35.7

### Donor ovarian stimulation and oocyte collection

Controlled ovarian stimulation in six donors was carried out following an induction protocol consisting in the administration of urinary human follicle-stimulating hormone (Fostipur, Angelini Farmaceutica; Barcelona, Spain), combined with gonadotrophin-releasing hormone antagonist (Cetrotide, Merck-Serono; Madrid, Spain) for down-regulation. The ovarian response was mainly monitored with periodical transvaginal ultrasounds. When at least three follicles with a diameter equal to or greater than 17 mm, 0.4 mg of subcutaneous gonadotrophin-releasing hormone analogue (Decapeptyl, Ipsen Pharma; Barcelona, Spain) was administered as ovulation inducer. Thirty-six hours after GnRH agonist administration, COC (oocyte complexes cumulus-corona-oocyte) were retrieved by transvaginal ultrasound-guided follicular aspiration, and isolated in a pre-warmed buffered medium (G-MOPS, Vitrolife; Goteborg, Sweden). COCs were distributed in four-well dishes (no more than 3–4 oocytes per well) containing 650 μl of Fertilization Medium (CookMedical, Ireland) and incubated at 37 °C in an atmosphere of 6 % CO_2_.

### Preparation of semen sample

Semen samples were collected by masturbation after an abstinence period of 3–5 days and just after egg retrieval by donor ovarian pick-up. After liquefaction, the parameters analyzed included: volume, concentration and motility. The methodology and criteria for assessing semen quality were those established by the World Health Organization (WHO, 2010). Sperm selection was performed in 40 % and 80 % discontinuous density gradients using PURESPERM (Nidacon International AB, Sweden). After 20' centrifugation at 300 x *g*, the pellet was recovered, washed with 3 ml of Gamete Buffer (CookMedical, Ireland) and centrifuged again for 10 'at 500 x *g*. Finally, the supernatant was removed and Fertilization Medium (CookMedical, Ireland) was added, adjusting the volume according to on the pellet recovered. The samples were incubated at 37 °C and 6 % CO_2_ until insemination [61].

### Insemination

The semen sample was first adjusted to concentration of 3x10^6^ spermatozoa per milliliter. Three hours after ovarian pick-up each well containing oocytes was inseminated with approximately 150,000 spermatozoa per well, covered with 300 μl of paraffin oil and incubated at 37 °C and 6 % CO_2_ in Fertilization Medium (FM) for 16–20 h. Simultaneously, only spermatozoa were incubated in the same conditions. After incubation the viability sperm was determinated using eosin-nigrosin staining technique. The mean of percentage of viability sperm was 60 % in all the samples analyzed.

### Experimental design

Six different cases of conventional IVF were used in this study. A total of seventy-two COC were inseminated. In all the cases the culture medium was FM. Four groups of media were used for metabolomics analysis and five groups of spermatozoa for determination of the AR. These groups were established as follows:

1. Eighteen aliquots (150 μl per aliquot) of medium from the wells containing only COC were collected just before insemination (immediately after 3 h of incubation), taking care to do not aspirate oocytes (*MBI: medium before insemination*).
2. Sperm obtained after density gradient centrifugation and 3 h incubation in FM at 37 °C and 6 % CO_2_ was used to assess AR *(CS:* control spermatozoa) for determination AR.
3. After assessing fertilization 16–20 h post-insemination, avoiding as far as possible aspirating dispersed cumulus cells, a total of eighteen aliquots (150 μl) of medium were obtained by centrifugation at 600g for 10 min, and the supernatant was used for metabolomic analysis (*MAI: medium after insemination*)*.* The pellets were used to analyze the percentage of acrosome reacted sperm *(SAI: spermatozoa after insemination).*
4. After 16–20 h incubation, the medium (150–200 μl) from the wells containing only spermatozoa was collected. After centrifugation at 600g for 10 min the supernatant was used for metabolomic analysis *(MOS: medium only spermatozoa),* and the pellets were used to analyze the percentage of acrosome reacted sperm *(SWI: spermatozoa without insemination).*
5. The FM incubated for 16–20 h at 37 °C and 6 % CO_2_ was also collected *(FM: control group for metabolomic analysis).*

Two additional groups for AR estimation were included to evaluate the effect of the cumulus cells alone or the conditioned medium where cumulus cells were present.

### Incubation of sperm with the cumulus cells in FM

Three hours after ovarian pick-up, 3 to 4 COC from 3 different donors were denudated by enzymatic digestion in hyaluronidase (80 IU/ml) followed by mechanical denudation by gentle pipetting in G-MOPS (Vitrolife). Media containing cumulus cells from each donor was transferred to conical tubes and then centrifugated at 300 g for 5 min. Pellets with cumulus cells were incubated in 500 μl FM (Cook) at 37 °C and 6 % CO_2_ in four-well dishes and inseminated with approximately 150,000 spermatozoa per well. One well containing only 500 μl of FM at 37 °C and 6 % CO_2_ was inseminated in the same conditions, as control. After 17–20 h, media containing spermatozoa from each well was transferred to conical tubes and then centrifuged at 600 g for 10 min.

### Incubation of sperm in supernatant of cumulus cells

Cumulus cells from 3 to 4 COC from one donor were recovered as described above. Pellet with cumulus cells were incubated in 500 μl FM (Cook) 37 °C and 6 % CO2 in a four-well dish for 17–20h. The supernatant obtained after centrifugation at 600 g for 10 min was inseminated with approximately 150,000 spermatozoa per well and incubated 37 °C and 6 % CO_2_. After 17–20h, media containing spermatozoa was transferred to conical tubes and then centrifuged at 600 g for 10 min. Pellet containing the sperm was used to evaluate the percentage of acrosome reacted sperm as previously described.

### Determination of the Acrosome Reaction (AR)

Percentage of acrosome reacted sperm was evaluated in the resuspended pellet obtained for the different experimental condition. Spermatozoa were fixed in 100 % methanol for 30 min at room temperature. Sperm aliquots (15 μl) were smeared onto glass slides and air-dried. The percentage of acrosome-reacted sperm was estimated according to the fluorescence pattern of their acrosomes using fluorescein isothiocyanate-labeled *Pisum sativum* agglutinin (FITC-PSA), as previously reported [62, 63]. The fluorescence patterns of 200 spermatozoa in randomly selected fields of each slide were determined using a Leica TCS SP2 (Leica Microsystems GmBH, Wetzlar, Germany) laser confocal microscope with x1,000 magnification. The acrosomal status of spermatozoa was classified according to the lectin staining as follows: (1) intact acrosome: complete staining of acrosome; (2) acrosome reacted: complete staining of equatorial segment only or no staining of the sperm head. The proportions of the two patterns were expressed as percentages of the total number of spermatozoa counted. The values of acrosome reaction in the three groups established (CS, SAI and SWI) passed the Kolmogorov-Smirnov normality test (K-S test, *P* > 0.05). Sources of significant variation were then assessed by one-way analysis of variance (ANOVA) and multiple comparisons using Tukey's Honest Significant Difference test (Tukey´s HSD). Descriptive (mean ± SE) and statistical analyses were conducted using SPSS v. 15.0 at *P* < 0.05 significance level.

### Metabolomic analysis

#### Commercial standards and reagents

The theobromine used as quality control compound for the metabolomic analysis was purchased from Sigma-Aldrich (Steinheim, Germany) and MS grade solvents such as water, acetonitrile, methanol and formic acid were purchased from Baker (Deventer, Netherlands).

#### Sample collection

Samples were analyzed taking into account the experimental design (double-blind and randomized).

#### Sample preparation

On the four groups established (MBI, MAI, MOS and FM), 2 mL of medium were prepared for protein precipitation with 3 mL of cold methanol (kept overnight at −20 °C) and vortexed to mix them. The samples were sonicated for 10 min in a 5510 Branson ultrasonic water bath (Bransonic, Danbury, USA). The samples were vortexed and then sonicated for a further 10 min. The proteins were pelleted by centrifugation at 4500 rpm (Sigma 1–13, B. Braun Biotech International, Osterode, Germany) for 10 min at room temperature. Subsequently, the supernatant was transferred to a vial and evaporated to dryness in a SpeedVac Concentrator Savant SPD 121 P (Thermo Scientific, Massachusetts, USA). The samples were reconstituted in 200 μL (v/v) of water: methanol (50:50) and each sample was vortexed, filtered and transferred to a glass vial for HPLC-q-TOF analysis.

#### HPLC-q-TOF analysis

Chromatography separation was performed on an 1100 series HPLC system (Agilent Technologies, Waldbron, Germany) equipped with on-line degasser, auto-sampler, quaternary pump, and thermostatic column compartment. An ACE 3 C18: 150 x 0.075 mm, 3 μm column (United Kingdom) were used. The mobile phase consisted of (A) H_2_O 0.1% HCOOH and (B) acetonitrile 0.1 % HCOOH. The injection volume was 6.25 nL and the flow rate was 312 nL/min for the culture media samples and quality controls (QCs). A gradient with the following proportions (v/v) of phase B (t, % B) was used for the determination of metabolites (0, 0); (0, 1); (10, 10); (11, 10); (17.5, 100); (19.5, 100); (19.6, 0); (23, 0). The HPLC was coupled to a quadrupole time-of-flight mass spectrometer (MS-q-TOF) (Bruker Daltonics, Bremen, Germany).

MS acquisition was performed by a Bruker MicroTOF-Q spectrometer (Bruker Daltonics, Bremen, Germany). Electrospray (ESI) analyses were carried out in positive and negative ion mode, with capillary and end plate offset voltages of −4500 and −500 V in positive mode, and 4000 and −500 V in negative mode. Nitrogen was used both as nebulizer and drying gas. The nebulizer gas pressure was 1.6 Bar, the drying gas temperature 200 °C and its flow rate 8.0 L/min. Spectra were acquired in the m/z 50–1200 range. In order to calibrate the mass axis, a 10 mM sodium formate solution in 1:1 isopropanol-water was introduced into the ESI source at the beginning of each HPLC run using a divert valve.

Two classes of quality control were used for the metabolomic analysis. QCs consisted to MS grade water samples and theobromine solution (20 μM) injected at three different times in the batch: beginning, middle and end. The samples were randomly ordered for injection.

Bruker Daltonics software packages micrOTOF Control v.2.3, HyStar v.3.2 and Data Analysis v.4.0 were used to control the MS(QTOF) apparatus, interface the HPLC with the MS system and process data, respectively.

#### Data processing

LC-MS data were analyzed by Profile Analysis software 2.0 (Bruker Daltonik, Bremen, Germany), which provides all the tools required for data statistical analysis. Raw data from LC-MS were transformed into a tabular format for their better management. Each data point is described by its retention time (RT) and *m/z* value called buckets with the corresponding intensities. Advance bucketing was performed in each analysis using the Find Molecular Feature (FMF) algorithm and the results were written into the data matrix, to reduce the enormous size of the LC-MS data.

In our analysis, the retention time and mass range were (0.02; 23.04 min) and (50; 900 Da), respectively. The bucket intensity values were normalized according to largest bucket value in all the samples and were used 50 % as buckets filter. This normalization step is important to ensure the comparative parameters among the different samples. The parameters controlled by FMF compound detection were S/N (signal to noise) threshold, 5; minimum compound length, 10; and smoothing width, 1. As regards general MS parameters, the spectrum type was linear and the spectrum polarity was obtained in the negative and positive modes in order to obtain the maximum number of data for cultures metabolome.

#### Multivariate statistical analysis

**Fig. 1:**
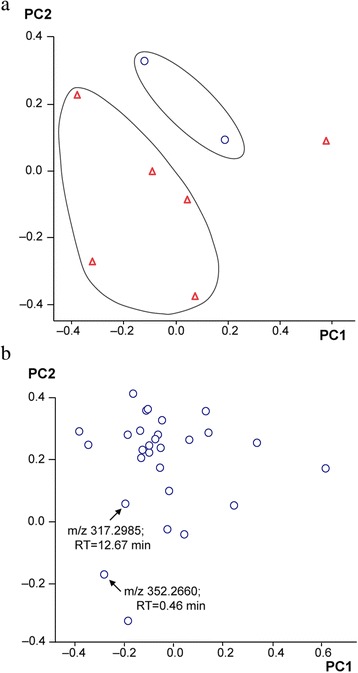
Principal Component Analysis (PCA) results. Score plot PC 1 *vs* PC 2 and (Data from cell samples (Δ) and control samples (O)) (**a**) (*PC*: principal component) and loading plot of PC 1 vs PC2 (**b**). Lower half of the loading plot shows some buckets discriminative (*m/z: mass/charge* and *RT: retention time*) in culture media samples. (ID: identity; *FM*: fertilization medium; *MBI*: medium before insemination; *MAI*: medium after insemination and *MOS*: medium only spermatozoa)

Principal Component Analysis (PCA) was performed using Profile Analysis after Pareto Scaling. PCA-based methods usually constitute the first step in evaluating metabolomic data. PCA is a tool used to reduce the dimensionality of a data set and allows the identification of the most influential variables. The new axes are called principal components (PCs). PC1 describes the largest variance in the data set. The variance explained is calculated as a sum of the individual variance values. In this context, PCA converts data obtained from LC-MS analysis into a visual representation: score plot (samples) and loading plot (buckets values) (Fig. 1). All samples were subjected to Student´s t-test, where a *P*-value < 0.05 was considered as significant.

#### Metabolite identification

Metabolites were identified on the basis of their exact mass, which was compared with those registered in various freely available databases like the Human Metabolome Database (www.hmdb.ca) and ChemSpider Database (www.chemspider.com).

Marker identification is possible thanks to a powerful analysis tool in the Profile Analysis software called SmartFormula, which provides information on the whole theoretical elemental composition for a particular m/z value. SmartFormula provided the empiric formula for the exact mass and the information on the isotopic pattern using a sigma algorithm (a combined value for standard deviation of the masses and intensities for all peaks). A mass tolerance value ≤ 5 mDa was used as complementary information to identify significant markers.

### Gene expression analysis

#### RT-PCR analysis in human cumulus oophorus

A gene expression analysis of human N-acylsphingosine amidohydrolase (acid ceramidase) 1 (ASAH1), human sphingolipid C4-hydroxylase (hDES2) and alkaline ceramidase 3 (ACER3) by RT-PCR in cumulus cells is performed to identify the potential contribution of the cumulus cells to the synthesis of the different metabolites. Cumulus cells from 13 COC were obtained from three additional patients. Total RNA was isolated using the RNeasy Mini Kit (Quiagen) according to the manufacturer’s instructions. The first-strand cDNA was synthesized from total RNA with the SuperScript First-Strand Synthesis System kit for RT-PCR (Invitrogen-Life Technologies), according to the manufacturer’s instructions.

ASAH1, hDES2 and ACER3 were partially amplified using the polymerase chain reaction (PCR) by means of specific primers. Two pairs of oligonucleotides were designed based on sequences deposited in GenBank (NG_177924, AY541700 and NM_018367, see Table 2). Amplifications were performed using 2 *μ*L of target cDNA, 0.5 *μ*g of each primer, 200 *μ*M of each dNTP and 1 U of Taq DNA polymerase. PCRs were carried out using an initial denaturation cycle of 2 min, and then 30 cycles of 95 °C for 1 min, 58 °C for 1 min and 72 °C for 1 min. The final extension time was 10 min at 72 °C. PCR products were analyzed by electrophoresis on 2 % agarose gels. Four microliters of the PCR reaction mixture were mixed with loading buffer and separated for 90 min at 100 V, before being visualized under UV light using ethidium bromide. Amplicons were carefully excised from the agarose gels and purified with a QIAquick Gel Extraction Kit Protocol (Quiagen), following the manufacter’s protocol. After that, the amplicons were automatically sequenced.

**Table 2:** Primers used in the amplification of ASAH1, hDES2 and ACER3

Gen (GenBank accession number)	Forward	Reverse	Amplified Region (bp)
ASAH1 (NM_177924)	catgtgaccgaacactgca	gttcaccatggttcgactg	250
hDES2 (AY541700)	gctggttcttctgcacac	ccttgaggaacatgtagtg	267
ACER3 (NM_018367)	gcttcttatttagcactcac	gcagatggtagtttactgag	172

### *In silico* analysis of the gene expression in cumulus cells and oocytes

Due to the complexity to obtain sufficient healthy metaphase II oocytes and to analyze by RT-PCR the PLA2 gene family an *in silico* analysis was performed. Thus, a detailed analysis of human RNA seq experiments stored in the Gene Expression Omnibus (GEO) accessible through GEO Series accession number GSE46490 [64], GSE36552 [65] and GSE44183 [66].

## Results

### Metabolomic analysis

The four groups of media (FM, MBI, MAI and MOS) were analyzed by LC-MS analysis for mass data collection. After peak alignment, a bucket table with approximately 1200 mass features was obtained. Principal Component Analysis (PCA) was used to produce interpretable directions of the samples in a reduced dimensionality and to reveal the buckets that influence group separation. PCA plots were used to determine metabolomic differentiation in the samples. A clear separation among the different culture medium samples can be observed (Fig. 1 (a)). Two first principal components (PCs) extracted accounted for 67.7 % of total variance of the LC-MS dataset (Fig. 1 (a)).

In this analysis eleven buckets were selected as the most significant, with *P* < 0.05 according to the m/z and retention time (Table 3). Three of them were identified in the positive mode as phytosphingosine (PHS), monoacylglyceride (MAG) and lysophosphatidylcholine (LPC), with a tolerance mass value of ≤ 5 mDa as additional parameter for the identification of the metabolites. PHS and MAG were detected in MAI and MOS groups. LPC was found in MBI, MAI and MOS groups; however, in the control group (FM), these metabolites were not detected. Fig. 1 (b) also depicts loading plot where significant buckets are far from the center (m/z 317.2985; 12.67min and m/z 352.2660; 0.46 min) and could be responsible for the variance within the data set. These metabolites were identified as monoacylglyceride, phospholipid and lysophospholipid classes.

**Table 3:** Identification of putative metabolites in culture medium samples. The metabolites are indicated according to the hierarchical order provided by a *t*-test at *P* < 0.05

	Buckets				Intensities			
ID	m/z	RT	Elemental Formula	Metabolite	FM	MBI	MAI	MOS
1	287.2845	13.48	C_14_H_39_O_5_	Unidentified	0	7197	5543	2802
2	293.9369	0.54	-	Unidentified	0	2812	2913	3076
3	300.1410	12.04	C_14_H_16_N_6_O_2_	Unidentified	0	2395	0	0
4	317.2985	12.67	C_18_H_39_NO_3_	Phytosphingosine	0	0	6224	4105
5	321.1973	2.67	C_13_H_27_N_3_O_6_	Unidentified	0	1766	2354	2263
6	352.2660	0.46	C_21_H_36_O_4_	Monoacylglyceride	0	0	514	448
7	408.1612	13.15	C_19_H_24_N_2_O_8_	Unidentified	0	20190	5593	6674
8	424.1354	13.17	C_23_H_16_N_6_O_3_	Unidentified	0	7432	0	2331
9	519.3386	12.33	C_26_H_50_NO_7_P	Lysophosphatidylcholine (18:2)	0	6211	4655	3166
10	735.8622	0.47	-	Unidentified	0	823	636	752
11	830.4411	9.79	-	Unidentified	0	2148	6736	11896

### Acrosome reaction

**Fig. 2:**
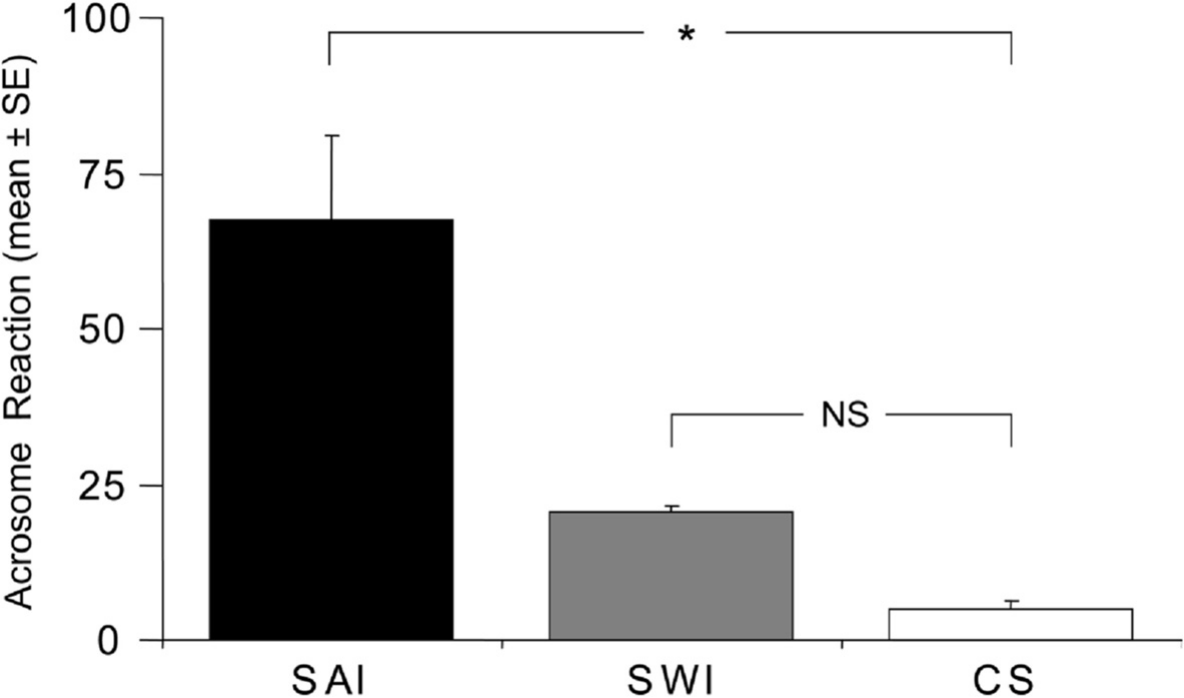
Differences in the percentage of acrosome-reacted spermatozoa between SAI, SWI and CS. Error bars indicate SE of the mean (% values). Asterisk denotes *P* < 0.05 (α-values maintained by sequential Tukey's HSD corrections). NS, not significant differences. SAI: spermatozoa after insemination; SWI: spermatozoa without insemination; CS: control spermatozoa

As described above LPC is able to induce the AR (de Lamirande et al., 1998) in human and bovine sperm. For that reason, the percentage of acrosome-reacted sperm was evaluated in the different experimental groups (Fig. 2). Significant spermatozoa variation was observed (F = 17.02; df = 2, 15; *P* < 0.001; ANOVA). Pairwise comparisons (Tukey´s HSD; *P* < 0.05) showed that the mean percentage value of acrosome-reacted sperm in the SAI experimental group (67.41 ± 13.572) was significantly higher (*P* < 0.01) than the values of both the SWI experimental group (20.67 ± 0.704) and CS experimental group (control group) (4.89 ± 1.249). In contrast, no statistical differences (*P* = 0.358) were found in the mean percentage of acrosome-reacted sperm when SWI and CS were compared.

The mean of percentage of acrosome reacted sperm after incubation of sperm with the cumulus cells in FM (39.3 ± 8.5 vs 18.5 ± 0.5) and supernatant of the cumulus cells (49.3 ± 1.5 vs 18.6 ± 1.5) was significantly greater (*P* < 0.05) than the control experiment.

### Gene expression analysis

#### Gene expression analysis of ASAH1, hDES2 and ACER3 by RT-PCR in human cumulus cells

**Fig. 3:**
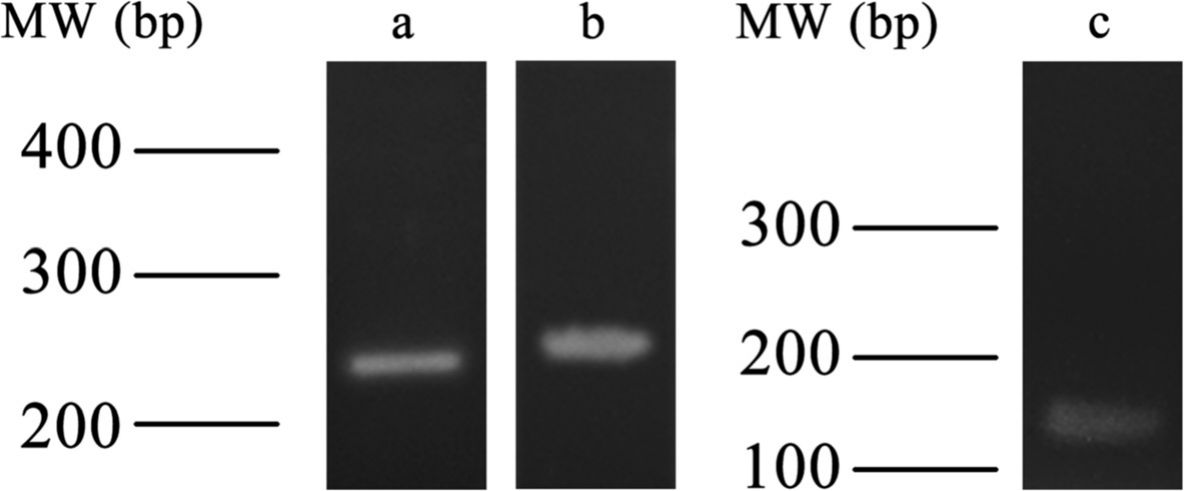
Amplicons corresponding to ASAH1, hDES2 and ACER3. 3.1 **a**) amplicon of 250 bp of ASAH1. **b**) amplicon of 267 bp of hDES2. 3.2 Amplicon corresponding to ACER3 (172 bp)

In order to know the source of the metabolites identified previously, the gene expression of different enzymes involved in their biosynthetic pathway was analyzed by RT-PCR analysis. cDNAs corresponding to ASAH1, hDES2 and ACER3 were partially amplified from the total RNA prepared from human cumulus cells (Fig. 3). Three amplicons of 250 bp, 267 bp and 172 bp were obtained corresponding to ASAH1, hDES2 and ACER3 respectively. These genes codify for three enzymes related with the sphingolipid synthetic pathways [67]. ASAH1 is a gene that encodes acid ceramidase. The protein catalyzes the synthesis and degradation of ceramide into sphingosine and fatty acid [68]. Furthermore, hDES2 exhibits a hydroxylase activity for dihydroceramide and reveals a high production of phytosphingolipids in different tissues [69]. ACER3 is an enzyme that hydrolyzes phytoceramide into PHS and free fatty acid [67, 70]. ACER3 and hDES2 gene expression in cumulus cells strongly suggests that these cells contribute to the formation of the PHS detected by metabolomic methods.

#### In silico gene expression analysis in human oocytes and cumulus cells

The *in silico* analysis of the gene expression that participate in the PHS synthesis was also performed in the human oocytes. Thus, a lower gene expression was observed with the ACER3 gene compared to the ASAH1 and hDES2 (Additional file 1: Table S1).

The *in silico* analysis of the gene expression allow to identify the expression of different gene of the PLA2 subfamily in cumulus cells (Additional file 2: Table S2). These genes are responsible of the synthesis of the LPC. The most expressed gene is the PLA2G16. In the human oocyte, two different studies indicate that the PLA2 gene expression is almost absent except for the PLA2G4C with a moderate expression however PLA2G12B is low (Additional file 1: Table S1) [65, 66].

## Discussion

Whether it occurs following the sperm binding to the ZP or during the sperm transit through the cumulus matrix, AR is a necessary event for the *in vivo* fertilization of the egg [10, 71]. Many different molecules present in the female genital tract, including steroids, phospholipids, small peptides and growth factors have been shown to induce AR [22, 72].

### Lysophosphatidylcholine

One of the metabolite found in our study was LPC. Phospholipase A2 (PLA2) metabolizes membrane lipids to arachidonic acid (AA) and LPC. Several works have described that LPC is able to induce the AR in different species, including human [49, 73–75]. The acrosome-reacted sperm produced by the LPC are fertile [73]. In 1986, Byrd and Wolf (1986) [75] indicated that the action of the LPC is very fast (15 min), leading to the rapid loss of motility (less than 2 min). Later, de Lamirande et al. (1998) [49] observed that 2.5 micromolar or below is not toxic, neither motility nor viability being affected. Therefore, the amount of LPC present in the oviductal fluid should be regulated precisely to avoid the above mentioned toxicity. No studies are available on the amount of LPC present in the oviductal fluid, although its presence in the follicular fluid and plasma serum has been reported. Thus, it was reported that the concentration of LPC is 286 mM and 252 mM in human follicular fluid and plasma, respectively [76].

A recent study [52] demonstrated that LPC is a better AR inducer than progesterone in bovine spermatozoa. In our study, LPC was first detected in the *in vitro* fertilization medium. LPC was detected with higher intensity in MBI and MAI (both medium contained COC), than in MOS (relative and semi-quantitative data). These results strongly suggest that cumulus cells contribute to LPC secretion. The LPC detected in MOS (medium with only spermatozoa) could be produced by these cells, which is consistent with the reports of other authors [77] concerning an increase of sperm LPC when the plasma membrane of the sperm is affected during acrosome reaction.

Moreover, LPC is able to induce the sperm AR, which is consistent with the higher percentages of acrosome-reacted human sperm observed after insemination (SAI group) when were incubated without COC (SWI group) (Fig. 2). The similar results obtained with isolated cumulus cells and conditionated medium obtained after the culture with cumulus cells support the previous finding. The smallest differences observed among the intact COC and the isolated cumulus cells and conditionated medium could be due to the abscence of different extracellular component present in the cumulus matrix (i.e. hyaluronic acid) or the differential gene expression due to the lost of gap junction among cumulus cells and/or oocytes. Further investigations will be required to study the kinetics of the AR induced by LPC and to compare the findings with those of a previous study [26], which used other AR inducers.

On the other hand, previous reports described how the stimulation of acrosomal exocytosis by some inductors leads to activation of PLA2 and the subsequent production of AA and lysophospholipids [78–80]. In addition, a recent study demonstrated that the PLA2 Group IID is present at the head and midpiece of human sperm where it plays a functional role during acrosomal exocytosis [81]. The PLA2 gene expression demonstrated by an *in silico* analysis of the cumulus cells (Additional file 2: Table S2) strongly supports that LPC is produced by the cumulus cells. Moreover, LPC at the time of fertilization could be involved in other important steps of fertilization in human as sperm-ZP binding and sperm-oocyte fusion as reported previously in rodents [82, 83]. Future experiments are necessary to test this hypothesis.

#### Phytosinphosine

Sphingolipids have emerged as essential secondary messengers in a variety of signal transduction pathways. Among these lipids, the ceramide is an important inducer of programmed cell death, i.e., apoptosis [84–86]. Previous studies demonstrated that the intestines, kidney and skin contained a considerable amount of phytosphingosine (PHS) [87–89]. On the other hand, Saisuga et al. (2012) [90], detected PHS in different tissues among them the testis, by a liquid chromatography/electrospray ionization tandem mass spectrometry method. PHS was detected in the MAI medium, when the COC and spermatozoa were incubated for 17 h. Additional experimental evidences support the presence of PHS in our study [91]. Thus, two different enzymes involved in the biosynthetic pathway of the PHS have been detected. DES2 was detected previously in other tissues in both mice [92] and human [69]. This enzyme is responsible for the synthesis of PHcer using DHcer as substrate. DHcer is the substrate of the ACER3 enzyme that produces PHS. The detection of the mRNA codifying for ACER3 in cumulus cells suggests that these cells are involved in the formation of PHS. Moreover, the *in silico* analysis showed that the ACER is expressed at high level in the cumulus cells [64] which is coincident with our data. However; the ACER3 gene expression in the human oocyte is very low suggesting a major role played by the cumulus cells compared to the oocyte [65, 66]. However, in the medium with only COC (MBI), in which the oocytes were incubated only for three hours PHS was not detected. This result could be explained by the short culture time used with this sample, and future experiments are necessary to confirm this hypothesis and to demonstrate a role of PHS as acrosome reaction inducer.

#### Monoacylglycerol

AR occurs during fertilization; O’Toole et al. (1996) [93] reported that human spermatozoa, stimulated with either progesterone or the Ca^2+^ ionophore A23187, undergo acrosomal exocytosis. This process entails the activation of phosphoinositidase C (PIC), and leads to hydrolysis of phosphatidylinositol 4,5-bisphosphate (PIP_2_) to generate inositol 1,4,5-trisphosphate (IP_3_), which stimulates the release of stored Ca^2+^, and diacylglycerol (DAG), which generally activates protein Kinase C [94].

In our case, only the monoacylglyceride was detected in the ESI. This was probably due to the spontaneous fragmentation of the pseudomolecular ion of diacylgliceride leading to the loss of an acyl moiety as a result of its weak bond like it is usually in the analysis of steryl fatty acyl esters by mass spectrometry [95]. This metabolite was found both in MAI and in MOS media containing acrosome-reacted sperm. No monoacylglyceryde was found in MBI this could be due to no sperm are present but also to short incubation time in this medium.

## Conclusions

**Fig. 4:**
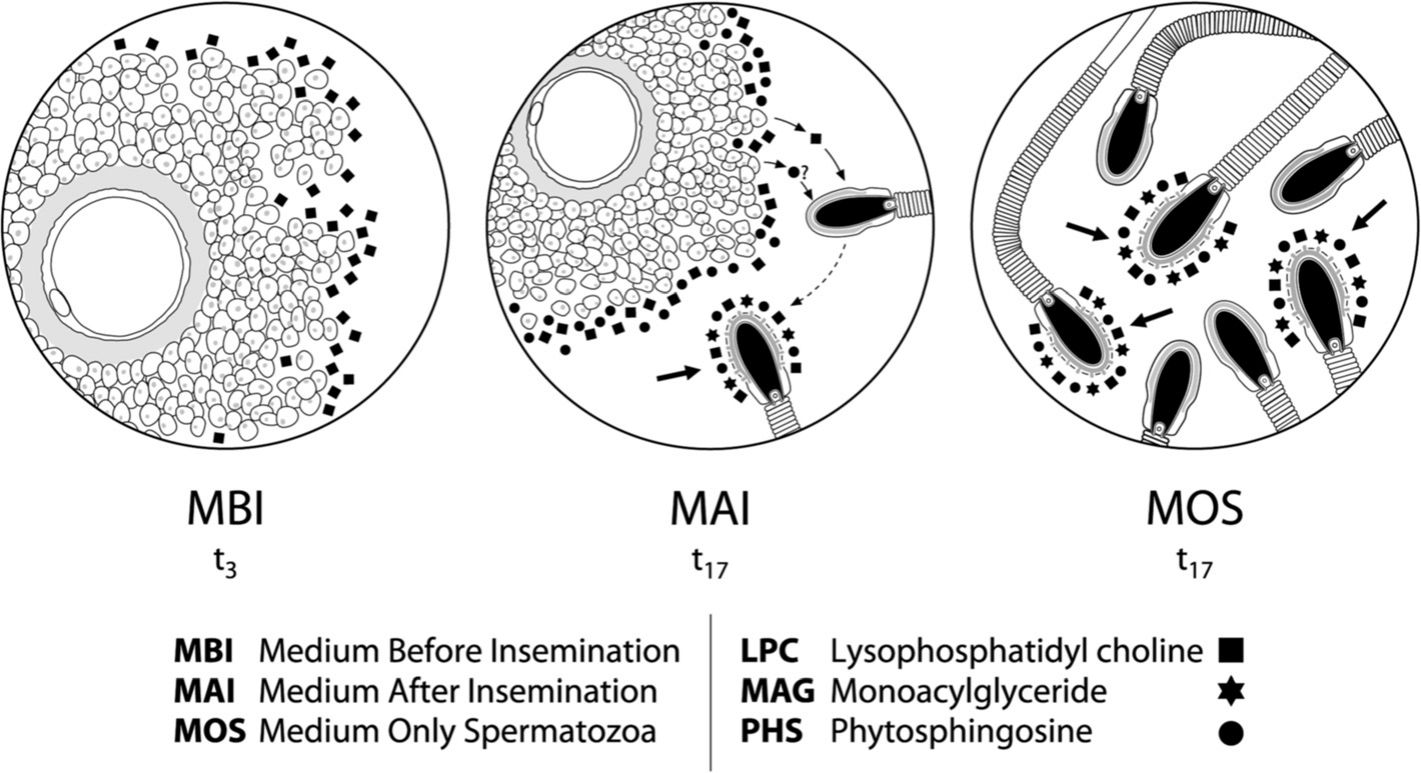
Working model for interplay of LPC/PHS/MAG metabolites during *in vitro* fertilization. Schematic diagrams showing: (**a**) Oocyte and cumulus cells culture during three hours (t3) and LPC secreted into MBI by cumulus cells. (**b**) Oocyte, cumulus cells and spermatozoa cultured during seventeen hours (t17) and LPC and PHS secreted by cumulus cells into MAI and inducing the acrosome reaction in spermatozoa. Acrosome reacted sperm (arrow) release LPC, PHS and MAG in the culture medium. (**c**) Only spermatozoa cultured for seventeen hours (t17). Spontaneous acrosome-reacted sperm are observed (arrows) which are probably responsible for the release of LPC, PHS and MAG into MOS

According to the metabolites found in this study, LPC and PHS are secreted by cumulus cells during culture (17 hrs). Both components could induce the AR in spermatozoa, contributing to the release into the medium of additional LPC, MAG and PHS by the acrosome-reacted sperm (Fig. 4). So, the identification of molecules with a paracrine effect in oocytes, cumulus cells and spermatozoa secreted during maturation and fertilization could help to improve the efficiency of ARTs thanks to a better understanding of the molecular dialogue between these specialized cells.

## Additional files

Additional file 1:
**Table S1.** Analysis of gene expression in human oocytes related with PHS and LPC synthesis (XLS 10 kb) Additional file 2:
**Table S2.** *In silico* analysis of the gene expression in cumulus cells related with PHS and LPC synthesis using the GSE46490 data (XLS 8 kb)
